# Revisiting secondary mitral regurgitation threshold severity: insights and lessons from the RESHAPE-HF2 trial

**DOI:** 10.1093/ehjopen/oeae084

**Published:** 2024-10-01

**Authors:** Patrizio Lancellotti, Tadafumi Sugimoto, Magnus Bäck

**Affiliations:** Department of Cardiology, University of Liège Hospital, GIGA Institutes, CHU Sart Tilman, Avenue de l'Hôpital, 1, 4000 Liège, Belgium; Department of Cardiology, Nagoya City University Mirai Kousei Hospital, 2 Chome-1501 Sekobo, Meito Ward, Nagoya, Aichi 465-0055, Japan; Department of Cardiology, Heart and Vascular Center, Karolinska University Hospital Solna, Anna Steckséns g 41, 17176 Stockholm, Sweden; Department of Medicine Solna, Karolinska Institutet, Anna Steckséns g 41, 17176 Stockholm, Sweden

**Keywords:** Secondary mitral regurgitation, Transcatheter edge-to-edge repair, Outcome

## Abstract

**Aims:**

This article revisits the severity threshold for secondary mitral regurgitation (MR), focusing on insights and lessons from the RESHAPE-HF2 trial. It aims to challenge the traditional effective regurgitant orifice area (EROA) threshold of ≥0.40 cm^2^ used for intervention, suggesting that earlier intervention may benefit patients with lower EROA. It also explores how transcatheter edge-to-edge repair (TEER) improves outcomes in patients with secondary MR and assesses the impact of left ventricular (LV) remodeling on treatment success.

**Methods and Results:**

The RESHAPE-HF2 trial evaluated the use of TEER in patients with moderate-to-severe secondary MR, comparing outcomes in those with an EROA ≥0.2 cm^2^ and no extensive LV remodeling. TEER significantly reduced heart failure hospitalizations and improved quality of life in these patients. This supports the notion that patients with less severe MR, who still show symptoms despite optimal medical therapy, may benefit from earlier intervention. Comparisons with COAPT and MITRA-FR trials underscored the importance of selecting patients based on MR severity relative to LV dilatation.

**Conclusions:**

The RESHAPE-HF2 trial highlights the need to reconsider the current EROA threshold for secondary MR intervention. TEER has shown to be beneficial even in patients with lower MR severity, suggesting that earlier intervention could improve outcomes. A more dynamic and integrated approach, considering both MR severity and LV remodeling, is essential for optimizing patient selection and treatment success.

Secondary mitral regurgitation (MR) often results from left ventricular (LV) remodelling, commonly due to ischaemic heart disease or dilated cardiomyopathy, or from atrial dilation related to conditions such as atrial fibrillation.^1,2^ Left ventricular remodelling leads to changes in the shape and function of the ventricle, which in turn causes the mitral valve to leak, worsening the overall heart function.^3^ Atrial dilation can also contribute by stretching the mitral valve’s supporting structures. As a result, blood regurgitates from the LV into the left atrium, worsening the haemodynamic burden on the heart.^4^ Clinically, secondary MR is associated with a poor prognosis, as it can exacerbate heart failure symptoms, promote further ventricular remodelling, and increase mortality.^5,6^ Therapeutic strategies often include optimal medical management of heart failure, with surgical or percutaneous interventions reserved for selected patients with severe MR and refractory symptoms.^7,8^

## The RESHAPE-HF2 trial

The RESHAPE-HF2 trial (Transcatheter Valve Repair in Heart Failure with Moderate to Severe Mitral Regurgitation), as the latest addition to the transcatheter edge-to-edge repair (TEER) research landscape, provides compelling new insights and raises important questions.^9^ This pivotal, investigator-initiated, prospective, randomized, multicentre study evaluated the impact of mitral valve TEER in patients with moderate-to-severe or severe secondary MR who were receiving maximally tolerated guideline-directed medical therapy. RESHAPE-HF2 found that mitral TEER significantly reduced the combined endpoint of heart failure hospitalizations and mortality compared with medical therapy alone. Compared with previous trials, the latter results are aligning more closely with the COAPT trial results than with MITRA-FR. Most of the benefit in RESHAPE-HF2 was linked to reduced hospitalizations (compared with 41 vs. 49% in COAPT), with notable improvements in quality of life, especially among those with recent heart failure hospitalizations (*Tables 1* and *2*).^10^

**Table 1:** Characteristics of the study populations in the randomized trials

	COAPT (*n* = 614)	MITRA-FR (*n* = 304)	RESHAPE-HF2 (*n* = 506)	MATTERHORN (*n* = 208)	STICH (*n* = 204)^a^
Trial intervention	TEER vs. control	TEER vs. control	TEER vs. control	TEER vs. Surgical	CABG vs. control
Age, years	72 ± 11	70 ± 10	70 ± 10	71 ± 8	61 ± 9
Sex (male)	64%	75%	80%	60%	82
Aetiology—ischaemic	61%	59%	65%	44%	100%
EuroSCORE II, median (IQR)	NR	6.6 (3.5–11.9)^b^	5.3 (2.8–9.0)	3.0 (1.7–4.3)	NR
6-min walk distance, m	240 (146–331)	310 ± 126^c^	292 ± 107	347 (240–400)	307 ± 113
ACEI or ARB or ARNI	67%	84%	82%	70%	NR
Beta-blocker	90%	90%	96%	84%	NR
Diuretics	89%	99%	95%	NR	NR
SGLT2 inhibitor	NR	NR	9%	10%	NR
NYHA III/IV	61%	67%	75%	86%	52%
NT-proBNP, pg/mL	5174 ± 6567^b^	3407 (1948–6790)^b^	4185 ± 4340 2745 (1407–5385)	NR	NR
eGFR, mL/min/1.73 m^2^	49 ± 26	50 ± 20	56 ± 21	57 ± 21	NR
LV ejection fraction, %	31 ± 9	33 ± 6	31 ± 8	43 ± 12	27 ± 8
LV end-diastolic volume, (mL/m^2^)	101 ± 34	135 ± 35	110 ± 40^d^	86 ± 30^d^	138 ± 46
EROA, cm²	0.41 ± 0.15	0.31 ± 0.10	0.25	0.20 ± 0.10	0.30^e^
Severe MR (EROA ≥ 0.4 cm^2^)	41%	16%	14%	NR	21%
Mortality, control group	2 years: 46.1%	2 years: 34.2%	2 years: 29.6%	1 year: 8.3%^b^	5 years: 55%^f^
All heart failure hospitalization, control group	2 years: 67.9 per 100 patients-years	2 years: 106.9 per 100 patients-years	2 years: 46.6 per 100 patients-years	1 year: 3%^b^	NR

ACEI, angiotensin-converting enzyme inhibitor; ARB, angiotensin receptor blocker; ARNI, angiotensin receptor–neprilysin inhibitor; CABG, coronary artery bypass graft surgery; eGFR, estimated glomerular filtration rate; IQR, interquartile range; LV, left ventricle; NR, not reported; NT-proBNP, n-terminal pro-B-type natriuretic peptide level; NYHA, New York Heart Association; SGLT2, sodium–glucose cotransporter 2.

^a^Subgroup of patients with moderate-to-severe mitral regurgitation.

^b^TEER group.

^c^ *n* = 223.

^d^Adjusted using the average body surface area in the COAPT trial.

^e^Extrapolated.

^f^Coronary artery bypass grafting alone.

**Table 2:** Impact of transcatheter edge-to-edge repair on primary endpoints in RESHAPE-HF2

	Impact on outcome	*P*-value
**Primary endpoints**		
Rate of heart failure hospitalizations or CV death (at 24 months)	36% ↓ in risk	0.002
Rate of recurrent heart failure hospitalizations (at 24 months)	41% ↓ in risk	0.002
KCCQ overall summary score (at 12 months)	11 points ↑ in QoL	<0.0001
**Secondary endpoints**		
NYHA Class I/II (at 12 months)	2.35 more likely	<0.0001
6-min walking test distance (at 12 months)	20.5 m ↑ performance	0.046

CV, cardiovascular; KCCQ, Kansas City Cardiomyopathy Questionnaire; NYHA, New York Heart Association.

## Comparing COAPT and MITRA-FR trials

The COAPT (Cardiovascular Outcomes Assessment of the MitraClip Percutaneous Therapy for Heart Failure Patients With Functional Mitral Regurgitation)^11^ and MITRA-FR^12^ trials are two landmark studies that evaluated the efficacy of TEER in patients with secondary MR. However, these trials yielded seemingly conflicting results, largely due to differences in patients’ selection and trial design. The COAPT trial demonstrated a significant benefit of TEER in reducing hospitalizations for heart failure and improving survival among patients with severe secondary MR. Importantly, the COAPT trial enrolled patients with more severe MR relative to their LV dysfunction. The patients had a higher effective regurgitant orifice area (EROA) and smaller LV volumes, indicating disproportionate MR.^13^ This meant that the MR was a primary driver of their symptoms, making them ideal candidates for MR intervention. In contrast, the MITRA-FR trial did not show a significant benefit of TEER over medical therapy alone. Patients in MITRA-FR had less severe MR relative to the degree of LV dysfunction, with larger LV volumes and a lower EROA. This suggested that in these patients, the MR was more a consequence of the underlying LV dilatation rather than a significant contributor to their heart failure symptoms.^14,15^ Therefore, treating the MR did not result in the same improvements seen in COAPT. The key discrepancy between COAPT and MITRA-FR lies in the ‘proportionality’ of MR to LV dysfunction. COAPT’s success was attributed to selecting patients where MR was a major contributor to heart failure symptoms (disproportionate MR), while MITRA-FR included patients where MR was more of a secondary effect of advanced LV disease (proportionate MR).^13^ This understanding has underscored the importance of precise patients’ selection for TEER therapy, particularly emphasizing the significant benefits for those with severe MR that is disproportionate to their degree of LV dysfunction. Consequently, the recent guidelines from the European Association of Cardiovascular Imaging have updated their severity thresholds for secondary MR to align with those for primary MR.^7^ These updated criteria now include an EROA of ≥0.40 cm² and/or a regurgitant volume (R Vol) of ≥60 mL, with adjusted considerations for crescent-shaped regurgitant orifices—specifically, an EROA ≥ 0.30 cm² and/or R Vol ≥ 45 mL in low-flow states—reflecting the complex nature of MR assessment.^1^

## RESHAPE-HF2 results: a less severe patient population

The RESHAPE-HF2 trial enrolled patients who were generally less ill compared with those in COAPT and MITRA-FR, as indicated by lower concentrations of natriuretic peptides, higher estimated glomerular filtration rate values, the exclusion of right ventricular dysfunction or severe pulmonary hypertension (which were not excluded in MITRA-FR), and a lower severity of secondary MR (mean EROA of 0.25 cm², compared with 0.40 cm² in COAPT and 0.31 cm² in MITRA-FR).^16^ In the MITRA-FR trial and RESHAPE-HF2, only 16 and 14% of patients, respectively, had severe MR (EROA of ≥0.40 cm²), compared with 41% in the COAPT trial. Patients in the RESHAPE-HF2 trial had a mean LV end-diastolic volume (EDV) of 211 mL, falling between the volumes observed in COAPT (194 mL) and MITRA-FR (252 mL). Unlike COAPT, the MITRA-FR trial did not exclude patients with significant LV dilation, with 70% of patients having an LV end-diastolic diameter exceeding 65–70 mm. In the RESHAPE-HF2 trial, only 33% of patients had an LV EDV >227 mL, reflecting a less advanced degree of LV remodelling compared with MITRA-FR.

## Evolving understanding of mitral regurgitation severity

The RESHAPE-HF2 trial underscores the complex interplay between patient characteristics, MR severity, and clinical outcomes, highlighting the need for nuanced management of secondary MR. This trial’s observations, notably the benefits manifesting primarily as reductions in hospitalizations, suggest that MR within this cohort was of less severe nature. However, these findings also prompt a reassessment of the conventional threshold for MR severity, historically set at an EROA of 0.2 cm², based on observational studies, which marks a critical turning point for significant prognostic implications.^3,5,17–21^ This threshold was later abandoned in the 2021 ESC guidelines, highlighting the evolving understanding of MR severity in clinical practice. Practically, it is imperative to view the progression of MR in conjunction with LV remodelling as a continuum. In clinical practice, the timing of intervention and the selection criteria for TEER are critical and should meticulously consider the degree of LV remodelling. When the LV EDV exceeds 200–220 mL (diameter > 65–70 mm), the pathophysiological burden of the underlying myocardial disease begins to dominate the clinical picture, reducing the relative impact of MR on symptoms and significantly diminishing the effectiveness of isolated mitral valve interventions.^9,21,22^ This threshold underscores the importance of evaluating MR severity in relation to LV volume, as ventricular size plays a crucial role in determining the success of interventions. Excessive LV dilation can diminish the effectiveness of MR correction, highlighting the need for timely intervention before severe remodelling occurs, when the potential for ventricular recovery is significantly reduced.^23^

## Mitral regurgitation severity and left ventricular remodelling: key factors in treatment success

**Figure 1:**
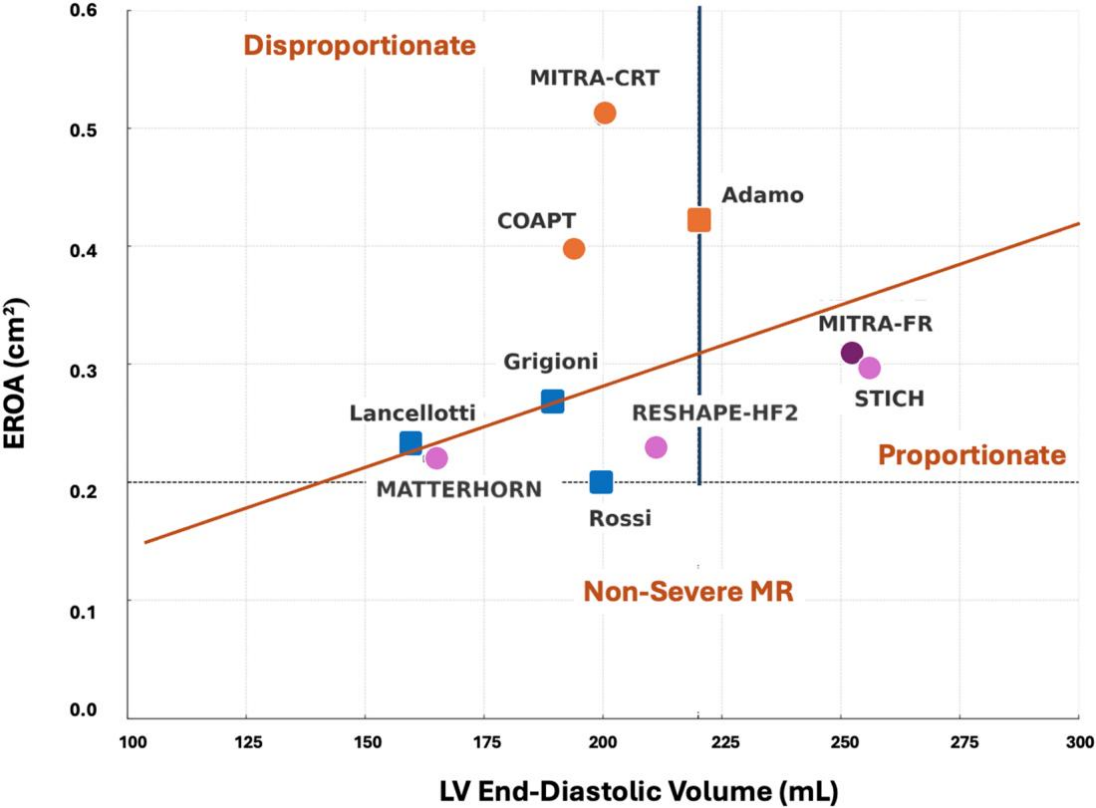
Ventricular end-diastolic volume vs. effective regurgitant orifice area across various studies. The red line represents a theoretical threshold designed to differentiate between disproportionate and proportionate mitral regurgitation relative to left ventricular size (from Grayburn *et al*.^13^). It helps identify potential responders to transcatheter edge-to-edge repair , with patients below the line more likely to experience improvements in quality of life and symptom relief, along with reduced heart failure hospitalizations, while those above the line, in the absence of extreme left ventricular remodelling, may benefit from improved survival. However, this remains a hypothesis, and there could be other forms of relationships that warrant further exploration in future studies. Randomized trials are represented by circles (orange for severe mitral regurgitation, magenta for moderate mitral regurgitation with positive outcomes from transcatheter edge-to-edge repair, or mitral valve repair in the case of Surgical Treatment for Ischaemic Heart Failure, with the exception of MITRA-FR, shown in dark magenta). The blue squares denote non-randomized observational studies, demonstrating the prognostic impact of moderate mitral regurgitation (effective regurgitant orifice area ≥0.20 cm²; Grigioni *et al.*,^3^ Rossi *et al*.,^18^ Lancellotti *et al.*^20^). The orange square represents a non-randomized study (Horii *et al.*^23^) that showed no benefit from transcatheter edge-to-edge repair in the presence of significant left ventricular remodelling.

*Figure 1* provides a nuanced comparison of LV EDV/EROA across several pivotal clinical studies that explore the implications of MR severity within diverse heart conditions. By plotting LV EDV against EROA, the graph illustrates the critical relationship between the extent of ventricular dilation and the severity of MR. Each category sheds light on different facets of managing MR, underscoring the complexity and varied approaches required for effective treatment. Patients who do not meet the COAPT trial criteria, particularly those with significant LV dilatation (LV EDV > 200–220 mL), generally derive no benefit from TEER.^22^ In the MITRA-CRT trial, patients with dilated cardiomyopathy and cardiac resynchronization therapy (CRT) non-responders experienced fewer cardiovascular deaths, heart transplants, and congestive heart failure hospitalizations with TEER compared with medical treatment alone.^24^ Despite enlarged LV, outcomes were still within the range promising positive results, akin to those observed in the RESHAPE-HF2 trial. The Surgical Treatment for Ischaemic Heart Failure (STICH) trial demonstrated that mitral valve surgery alongside cardiac surgery significantly reduced long-term mortality in patients with LV systolic dysfunction and coronary artery disease.^25,26^ Notably, the LV end-diastolic dimensions and degree of MR in STICH patients resembled those in MITRA-FR, and survival benefits were akin to those observed in the COAPT trial. The observed differences with MITRA-FR can be attributed to the potential for myocardial recovery following revascularization and mitral valve surgery. The MATTERHORN trial demonstrated that TEER achieved non-inferiority to surgical intervention at the 1-year composite endpoint in patients with heart failure with moderate secondary MR.^27^ Remarkably, it also showed a 1-year mortality rate of 8.3%, significantly lower than the 15–35% typically seen in medically treated patients with an EROA exceeding 0.2 cm²,^3,16,19,20^ underscoring TEER’s substantial prognostic advantage in this setting.

## Conclusion

Considering the recent RESHAPE-HF2 results, there is a compelling need to revisit the traditional severity threshold for secondary MR, specifically the EROA of ≥0.40 cm² used for referring patients for intervention in the ESC guidelines. This threshold appears increasingly questionable, as emerging evidence suggests that patients with lower EROA values may also benefit from earlier intervention. Transcatheter edge-to-edge repair has shown promise as a viable therapeutic option to improve quality of life and symptom relief, as well as reduce heart failure hospitalizations, in patients with an EROA >0.2 cm² and no significant LV remodelling (LV EDV < 200–220 mL) who remain symptomatic despite optimal medical treatment. However, these cases present significant clinical challenges, as management remains contentious. A more nuanced, integrated approach that considers both static and dynamic changes in MR severity may be crucial for refining treatment decisions.^19^ The dynamic nature of MR has not been examined in these studies, potentially affecting the interpretation of results. Additionally, it is essential to further understand how LV remodelling influences TEER outcomes and to explore the impact of emerging treatments, such as sodium–glucose cotransporter 2 inhibitors, which were underutilized in these trials. These insights could play a key role in shaping future management strategies for secondary MR, highlighting the need for a dynamic and adaptive therapeutic approach.

## Data Availability

There are no new data associated with this article.
